# Modulation of cellular signaling and innate immune mechanisms by cold atmospheric plasma

**DOI:** 10.3389/fimmu.2026.1926559

**Published:** 2026-09-04

**Authors:** Fawad Khan, Michael P. Schön

**Affiliations:** Department of Dermatology, Venereology and Allergology, University Medical Center Göttingen, Göttingen, Germany

**Keywords:** cold atmospheric plasma, immunogenic cell death, immunomodulation, infection control, macrophage polarization, plasma medicine, reactive oxygen/nitrogen species, redox signaling

## Abstract

Cold atmospheric plasma (CAP) has emerged as a tunable physicochemical stimulus capable of modulating immune responses through the controlled delivery of reactive oxygen and nitrogen species (RONS), charged particles, transient electric fields, and photons at near-ambient temperature. While the antimicrobial and cytotoxic applications of CAP are well established, evidence regarding its role as a regulator of innate immune signaling remains fragmented across the literature. Inconsistent dosimetry reporting is a noted issue, and a unified framework linking plasma-generated redox signals to specific immune cell outcomes is lacking. This narrative review focuses on the effects of CAP on innate immune mechanisms and the cellular signaling pathways that translate plasma-derived redox cues into biological responses. We first outline how device architecture, gas composition, treatment mode, and dose shape the qualitative and quantitative RONS profile that reaches biological targets. We then discuss key redox-sensitive signaling pathways, including NF-κB, Nrf2-Keap1, MAPK, PI3K-AKT-mTOR, HIF-1α, mitochondrial pathways, and inflammasome activation. While NF-κB, Nrf2-Keap1, and MAPK signaling are supported across multiple independent studies, evidence for HIF-1α and specific inflammasome subtypes is limited and derived from a smaller number of experimental models. Particular emphasis is placed on macrophage polarization, where available evidence indicates that CAP may promote either proinflammatory, antimicrobial M1-like programs or anti-inflammatory, tissue-repairing M2-like phenotypes depending on exposure parameters and tissue context. We further summarize more limited and still-emerging evidence that CAP influences dendritic cell maturation, antigen-presenting capacity, neutrophil migration and neutrophil extracellular trap (NET) formation, and natural killer (NK) cell-mediated tumor-cell recognition. Collectively, current evidence indicates that CAP is not merely a cytotoxic or antimicrobial modality but a potential dose- and context-dependent regulator of innate immune function, though findings vary across plasma sources and experimental models. A deeper understanding of CAP dosimetry, RONS chemistry, and cell-type-specific redox thresholds will be essential for facilitating future clinical translation into applications in infection control, wound healing, inflammation, and oncology.

## Introduction

Cold atmospheric plasma (CAP), an ionized gas that is sustained at atmospheric pressure and room temperature, has emerged as a promising technology reported to modulate a wide range of biological functions (1). Low-temperature plasma consists of a mixture of chemically reactive species, including reactive oxygen species (ROS) and reactive nitrogen species (RNS), which are established mediators of redox biology and regulators of numerous cellular and biological processes (2, 3). Together, these species are referred to as reactive oxygen and nitrogen species (RONS).

Beyond its well-documented antimicrobial and anticancer effects, CAP is increasingly recognized as a candidate tool for modulating immune cell functions, with potential roles in shaping inflammation, host defense, and antitumor immunity. Initial studies revealed that CAP exerts direct bactericidal activity and cytotoxic effects on cancer cells, which are largely attributable to short-lived ROS, such as hydroxyl radicals and singlet oxygen (4–6). Subsequent work highlighted that sub-lethal CAP exposures modulate redox-sensitive signaling cascades in keratinocytes (7–10), immune (11), and stromal cells (12), with reported effects on cytokine expression (13, 14) and antigen presentation (15–19). These discoveries place CAP firmly within the growing field of “plasma medicine”, where physicochemical stimuli are harnessed for therapeutic purposes (20).

We focus specifically on innate immune mechanisms for three reasons. First, innate immune cells, including neutrophils, macrophages, dendritic cells (DCs), and natural killer (NK) cells, are typically the first responders at CAP-treated surfaces (e.g., skin, chronic wounds, and the tumor microenvironment), and are thus among the cell types most directly exposed to plasma-derived RONS (21–23). Second, the redox-sensitive signaling pathways governing innate immune activation (e.g., NF-κB (nuclear factor kappa-light-chain-enhancer of activated B cells), Nrf2-Keap1 (nuclear factor erythroid 2-related factor 2-Kelch-like ECH-associated protein 1), inflammasome assembly) are comparatively well characterized in general redox biology, offering a mechanistic entry point for dissecting CAP-specific effects (24, 25). Third, innate immune modulation is increasingly proposed as a mechanistic bridge linking the direct antimicrobial/cytotoxic actions of CAP to downstream effects on adaptive immunity, wound repair, and antitumor responses, making it a logical focal point for a review aiming to clarify mechanism-to-outcome relationships (26).

At the cellular level, CAP has been reported to directly and indirectly alter the behavior of multiple immune cell populations, although the strength and consistency of evidence vary by cell type. In neutrophils, CAP or plasma-treated solutions alter migration and ROS production and enhance activation-marker expression without inducing apoptosis in several studies, suggesting a shift toward improved immunoreactivity rather than overt damage (21, 23). Monocytes and macrophages have been shown to undergo redox-dependent phenotypic shifts, with CAP driving polarization toward either M1 or M2 states depending on the context. CAP has also been reported to enhance bactericidal activity, potentially supporting both the resolution of inflammation and microbial clearance (27, 28). DCs respond to CAP and CAP-treated medium by increasing phagocytosis of dying tumor cells and maturation and by upregulating co-stimulatory ligands and proinflammatory cytokines, which may facilitate antigen presentation and priming of tumor-specific T cell responses (17, 19, 29–31).

Despite this expanding body of work, a significant knowledge gap remains. There is currently no integrated framework linking cold plasma engineering parameters, such as device architecture, gas composition, and dose, to the specific redox-sensitive signaling pathways activated in different innate immune cell types, nor is there consensus on how these pathways translate into functional phenotypic outcomes. Furthermore, dosimetry reporting varies considerably across studies, complicating cross-study comparison.

In this review, we examine the mechanisms underlying CAP-induced innate immunomodulation by integrating current evidence across plasma physics, RONS biochemistry, and immunology. We first describe how plasma device architecture, gas composition, and treatment parameters shape the RONS profile delivered to biological targets. We then discuss the major redox-sensitive signaling pathways, followed by a detailed evaluation of CAP’s effects on macrophage polarization, dendritic cell maturation, neutrophil function, and NK cell activity. We conclude by identifying unresolved questions and proposing future directions to support reproducible study design and the rational development of CAP-based applications in infection control, wound healing, inflammation, and oncology.

## CAP generation and device architectures

CAP is generated when a high electric field accelerates free electrons in a feed gas (air, He, Ar, or N_2_/O_2_ mixtures), initiating ionization cascades (32). Several device architectures have been developed to generate CAP for biomedical use, each with distinct advantages, limitations, and suitability for specific applications (**Table 1**).

**Table 1:** CAP-based device types.

Device type	Core structure/field	Typical strengths	Limitations	Primary biomedical suitability	References
DBDs	Two electrodes + dielectric barrier	Large-area, uniform, safe non-thermal plasma	Requires close proximity to target; limited for irregular/internal surfaces	Surface sterilization, wound healing, dermatology	(32, 33)
Plasma jet (APPJs)	Gas flow between electrodes, plume in air	Local, deep access, flexible geometries	Smaller area of treatment; RONS output sensitive to distance/air entrainment; higher gas consumption	Wound care, endoscopic treatment, oncology	(41, 142)
Microwave/radiofrequency plasma	High-frequency electromagnetic fields ionize gas	Efficient ionization, used in some food and industrial setups	Higher device complexity/cost; less adaptable to contoured surfaces	Large wound surfaces, large-area decontamination; less characterized; limited immunological data; chronic wound care (emerging), scalable treatment (emerging)	(37, 51)
Hybrid/emerging platforms	Combined jet-DBD or multi-jet arrays	Combines directional and uniform delivery; scalable	Less characterized; limited immunological data	Chronic wound care, scalable treatment (emerging)	(47, 48)

*Dielectric barrier discharges (DBDs)*: Planar or cylindrical electrodes separated by a dielectric produce surface-confined plasma with nanosecond microdischarges. In principle, a high-voltage alternating current (AC) source (kV, kHz range) is applied between two electrodes separated by at least one insulating (dielectric) layer. This configuration prevents the transition to a thermal arc discharge. Instead, it sustains numerous microdischarges, producing a non-thermal plasma (32–35). DBDs offer relatively simple construction, low gas consumption, and the ability to treat larger surface areas uniformly, making them attractive for surface sterilization, decontamination, wound healing, food preservation, and textile/polymer treatment (33, 36, 37). However, DBD configurations typically require close proximity between the electrode/dielectric assembly and the treated surface, limiting their applicability to irregular or internal anatomical sites. In addition, the direct proximity of the discharge to tissue raises considerations regarding transient electric field exposure and charge deposition (38). *Atmospheric-pressure plasma jets (APPJs)*: Plasma is ignited within a flowing gas stream (He, Ar, or air) using electrodes integrated into a dielectric tube; the reactive effluent exits as a directed plasma plume into ambient air, enabling treatment of targeted or difficult-to-access tissues, including in endoscopic applications (39–41). APPJs offer greater flexibility for localized and directional treatment and are primarily employed in medical applications, including wound care, dermatology, and oncology (42, 43). Their limitations include a comparatively smaller treatment area, sensitivity of RONS output to nozzle-to-target distance and ambient air factors, and generally higher operating gas consumption (particularly for noble-gas-fed jets), which may raise cost and logistical considerations for clinical use (44).

*Microwave or surface-micro-discharge arrays*: These configurations use high-frequency electromagnetic fields to generate large-area homogeneous plasma, making them suitable for uniform treatment of wound surfaces or large areas (45, 46). Trade-offs include increased device complexity and cost, and generally less flexibility for treating irregular or contoured surfaces compared to jet-based systems.

*Emerging and hybrid platforms*: Beyond these established architectures, several hybrid and next-generation systems have emerged, including combined jet-DBD hybrid configurations designed to merge the directional delivery of jets with the surface uniformity of DBDs, array-based multi-jet systems, and flexible or wearable CAP devices under development for continuous or repeated treatment of chronic wounds (47, 48). These platforms remain comparatively less characterized in the immunological literature and represent an area for future mechanistic studies.

Among these architectures, DBD systems and plasma jets appear most frequently represented in the literature in the context of CAP-immune cell interactions, with microwave/radiofrequency (RF) discharges serving as complementary technologies for specific large-area applications (49, 50). Generally, these devices can be tailored for broad-area treatment, highly localized plasma delivery, or patterned surface processing in medicine, food, and materials applications by adjusting electrode geometry, dielectric design, operating frequency, and gas conditions. The electron energy distribution function and, consequently, the composition and relative abundance of the RONS delivered to the target, are collectively determined by device geometry, power, frequency, and gas flow rates (32, 51, 52).

However, a significant challenge is the substantial variability in device design, operating parameters, and reporting practices across studies. Applied power, treatment distance, gas flow rate, exposure time, and electric field strength are inconsistently reported across the literature, complicating direct comparison of biological outcomes between studies and laboratories. This lack of standardization represents a major barrier to reproducibility and clinical translation of CAP-based immunomodulatory therapies. Importantly, the biological effect of CAP depends not only on device architecture but also on the delivered plasma dose, treatment time, distance, frequency of application, and resultant RONS flux at the target surface. Two devices of identical architecture can therefore produce divergent biological outcomes if operated under different dosimetric conditions, and conversely, different device types may be tuned to deliver comparable doses.

## Primary and secondary reactive species generated by CAP

**Figure 1:**
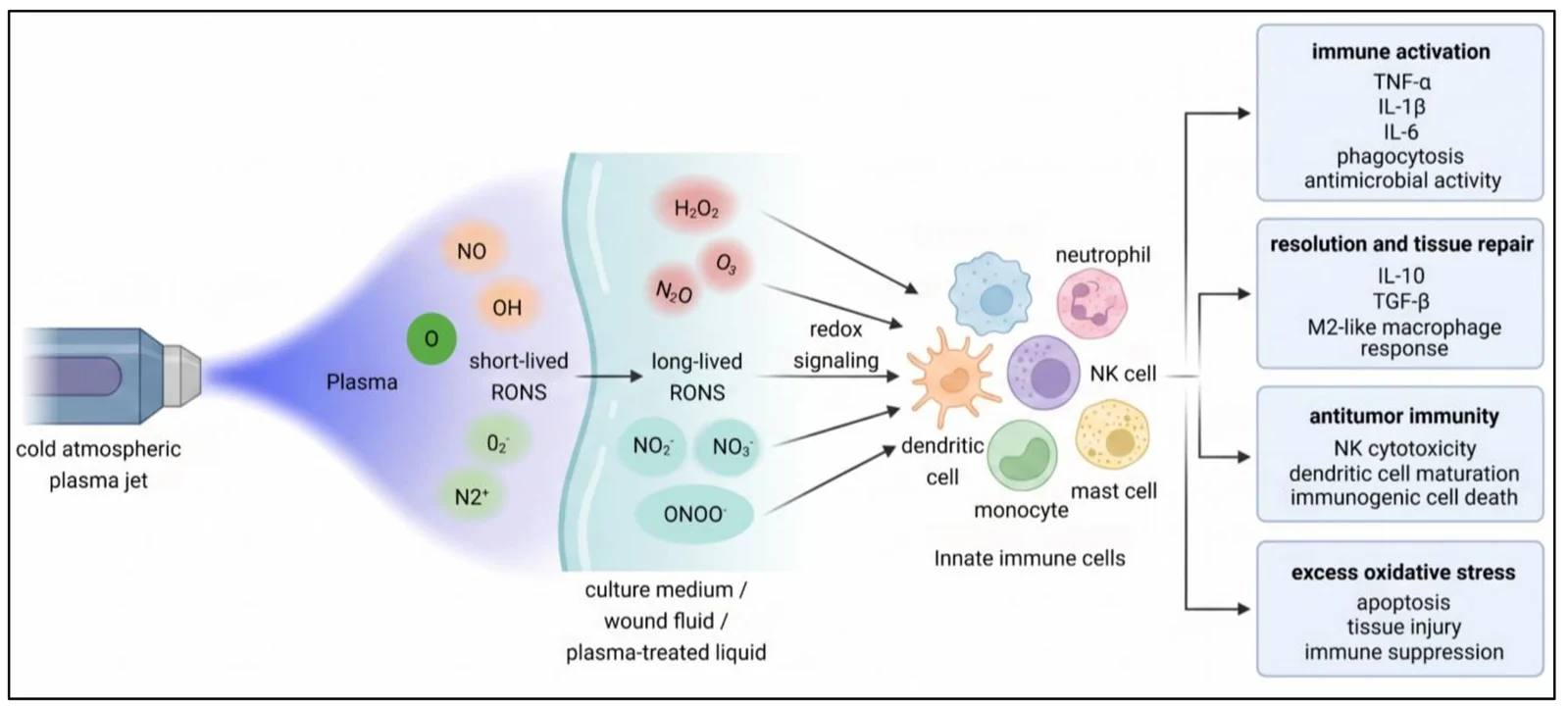
Cold atmospheric plasma and generation of biologically relevant RONS. CAP devices generate multiple reactive species, including short-lived and long-lived RONS, which interact with biological liquids to form secondary redox mediators capable of modulating innate immune cell functions. Depending on CAP dose, exposure conditions, cell type, and disease context, CAP can promote immune activation, antimicrobial defense, tissue repair, antitumor immunity, or oxidative injury.

CAP sources generate partially ionized gases at near-room temperature that are rich in reactive oxygen and nitrogen species. These species are produced through a sequential process: generation in the plasma core, transformation in the afterglow region, and final chemical evolution at gas-liquid or gas-tissue interfaces (**Figure 1**) (34, 39, 53, 54).

Primary species are directly generated within the plasma core predominantly through electron-impact dissociation, ionization, and excitation reactions. These include reactive atoms and short-lived radicals such as atomic oxygen (O), hydroxyl radical (OH), nitric oxide (NO), superoxide anion (O_2_^-^), excited molecular species (e.g., N_2_^+^, singlet oxygen ¹O_2_), free electrons (e^-^), and vacuum-ultraviolet (VUV)/UV photons. These species are formed within nanoseconds to microseconds in the plasma discharge itself, and generally possess very short lifetimes under atmospheric conditions (39, 53). These primary species react rapidly and non-selectively near their site of generation, and are therefore largely confined to the immediate treatment surface or the outermost liquid/tissue layer, where they can directly oxidize membrane lipids, surface proteins, and extracellular matrix components. Upon transport through the afterglow and subsequent contact with aqueous interfaces (cell culture medium, wound exudate, mucus), primary species undergo further chemical transformation to generate secondary, longer-lived species such as hydrogen peroxide (H_2_O_2_), nitrite (NO_2_^-^), nitrate (NO_3_^-^), ozone (O_3_), dinitrogen oxide species (N_2_O, N_2_O_5_), peroxynitrite (ONOO^-^), and carbonate radicals (20, 55–57). These secondary species are not generated directly within the plasma phase but instead predominantly arise from interfacial and aqueous-phase reactions, including dissolution of gas-phase O_3_, NO, and NO_2_ governed by Henry’s law solubility constants (58, 59), hydrolysis reactions, and radical-radical recombination (60, 61).

When dosed appropriately, these secondary species can diffuse across cell culture medium, wound exudate, or into deeper tissue layers, thereby enabling sustained and spatially distributed redox signaling. In the context of CAP-mediated immunomodulation, short-lived species are thought to contribute predominantly to direct antimicrobial and cytotoxic effects at the treatment surface, whereas long-lived species are more frequently implicated in the redox-sensitive signaling cascades (e.g., NF-κB, Nrf2-Keap1 activation) that lead to downstream immune cell responses (16, 62, 63).

The capacity of any given species to diffuse across biological barriers and engage intracellular redox signaling is not uniform, but depends on its chemical properties (charge, polarity, lipophilicity), half-life, local concentration gradient, and the specific barrier properties of the target tissue (e.g., mucus layer thickness and composition, epithelial junction integrity, local antioxidant capacity) (64, 65). Consequently, statements regarding transmembrane diffusion and redox signaling should be interpreted as species- and context-dependent rather than as a generalized property of CAP-derived RONS.

Both the primary and secondary RONS are strongly shaped by device architecture, feed gas composition, and operating parameters (34, 39, 53, 54). For example, He-fed plasma jets typically generate abundant stable He species that facilitate efficient ionization of ambient air, favoring generation of reactive oxygen species with comparatively lower reactive nitrogen output (40). In contrast, air-fed or N_2_/O_2_ gas mixtures, more commonly used in DBD configurations, promote substantially greater RNS output (NO, NO_2_, and downstream NO_2_^-^/NO_3_^-^) (66, 67). These device- and parameter-dependent differences in RONS qualitative and quantitative composition are directly relevant to the differential immune cell responses, underscoring the need for precise reporting of operating conditions in CAP-immunology studies.

Accurate characterization of CAP-generated RONS relies on a range of analytical techniques, each with distinct strengths, limitations, and species specificity. Primary species are commonly characterized using optical emission spectroscopy and mass spectrometry (68, 69), while aqueous-phase secondary species are typically quantified using fluorometric and colorimetric assays (e.g., Amplex Red for H_2_O_2_, Griess assay for nitrite) (70, 71), and electron paramagnetic resonance (72). Importantly, differences in analytical sensitivity, species specificity, and susceptibility to interference among these methods can lead to substantial variability in reported RONS concentrations across studies, complicating direct comparison of dosimetric and biological outcomes. Standardized reporting of the specific detection method, calibration approach, and detection limits is therefore essential for improving reproducibility and cross-study comparability in the field.

## Molecular pathways: redox signaling in immune cells

Cold atmospheric plasma generates RONS, which act as both stressors and signaling molecules in immune cells. These redox cues have been reported to suppress inflammation, enhance antimicrobial defense, or shape antitumor immunity, depending on dose, exposure mode, and microenvironment. The activation and fate of immune cells are tightly controlled by ROS/RNS levels and antioxidant systems, such as glutathione (GSH), thioredoxin (TRX), and Nrf2-dependent responses (24, 25, 73–75). Much of the mechanistic detail described below has been established in general redox biology and subsequently extended to CAP-specific contexts, where CAP-specific experimental validation is limited.

**Figure 2:**
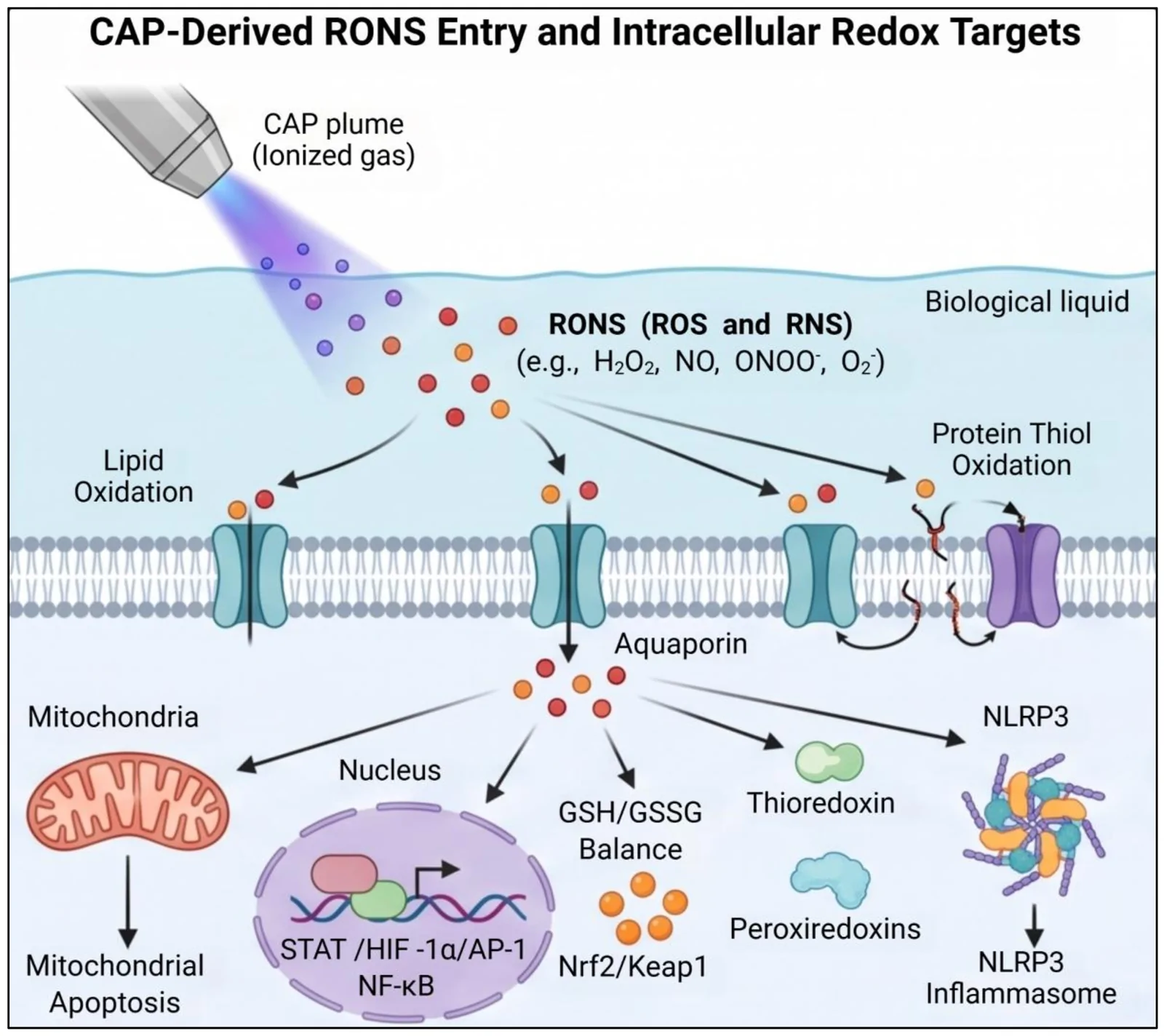
CAP-derived RONS entry and intracellular redox targets. CAP-derived RONS regulate multiple redox-sensitive signaling pathways in innate immune cells. RONS enter cells through direct diffusion, membrane lipid oxidation, or transport through channels such as aquaporins. Once inside the cell, CAP-derived RONS modulate redox-sensitive targets, including the Nrf2/Keap1 pathway. RONS can also influence nuclear transcription factors such as NF-κB, STAT, HIF-1α, and AP-1, and affect mitochondrial function and apoptosis. In immune signaling, intracellular redox changes may contribute to activation of the NLRP3 inflammasome. Overall, CAP-generated RONS regulate intracellular oxidative stress, antioxidant defenses, inflammation, and cell fate pathways.

Key redox sensors and transcription factors implicated in CAP-mediated immune modulation include NF-κB, Nrf2–Keap1, HIF-1α, PI3K-AKT-mTOR, MAPKs, and the NLRP3 inflammasome (**Figure 2**). These factors regulate macrophage/T-helper polarization, cytokine profiles, and immunogenic *versus* tolerogenic states (76–78), although the degree of direct experimental support varies by pathway. Indeed, modulation of NF-κB and Nrf2-Keap1 by CAP has been demonstrated across multiple independent studies and cell types, whereas HIF-1α (hypoxia-inducible factor-1α) and NLRP3 (nucleotide-binding oligomerization domain-like receptor family pyrin domain containing 3) involvement is comparatively less extensively validated in CAP-specific systems.

The conserved Cys62 in the NF-κB p50 subunit is redox-sensitive, and oxidative modifications (e.g., S-glutathionylation or sulfenic acid) at this site have been shown to inhibit the DNA binding of the p50 subunit. This, in turn, limits the NF-κB-mediated transcription of proinflammatory genes, such as TNF-α (tumor necrosis factor-α) and IL (interleukin)-6 (79, 80). In the Nrf2–Keap1 complex, the oxidative modification of Keap1 cysteines releases Nrf2, resulting in the upregulation of antioxidant genes, such as HO-1 (heme oxygenase 1; HMOX1) and NQO1 (nicotinamide adenine dinucleotide dehydrogenase [quinone] 1). These Nrf2-induced enzymes exert antioxidant and anti-inflammatory effects that can temper inflammatory signaling and cytokine production (81–83).

**Figure 3:**
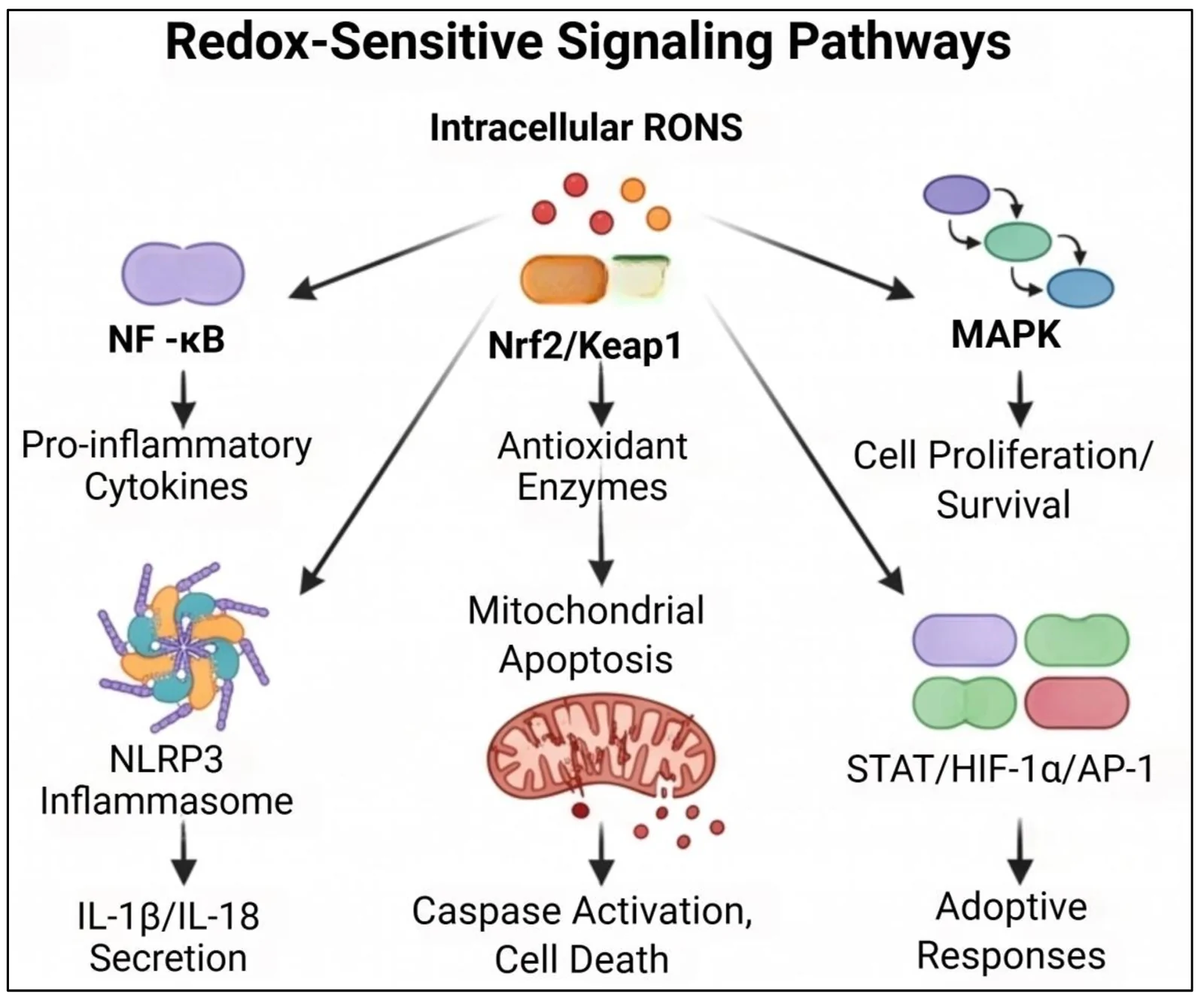
Redox-sensitive signaling pathways regulated by intracellular RONS. The presumed or already demonstrated direct interactions of RONS with intracellular mediators may explain a number of known biological effects of CAP. RONS can activate NF-κB, promoting the expression of pro-inflammatory cytokines, and stimulate the NLRP3 inflammasome, leading to IL-1β and IL-18 secretion. RONS also modulate the Nrf2/Keap1 pathway, inducing antioxidant enzyme expression to restore redox balance. At higher or sustained levels, RONS can disrupt mitochondrial function, triggering caspase activation and cell death. In parallel, RONS influence MAPK signaling, supporting cell proliferation and survival, and regulate transcriptional programs involving STAT, HIF-1α, and AP-1, contributing to adaptive cellular responses. All of these effects and their interactions with one another are highly context- and dose-dependent.

These pathways do not operate in isolation, and their crosstalk is increasingly recognized as central to the net immunological outcome of CAP exposure, although direct CAP-specific evidence for many of these interactions remains limited and is often extrapolated from general redox biology. Reports and proposed interactions include: i) Nrf2-mediated transcriptional and non-transcriptional suppression of NF-κB activity, whereby Nrf2 activation can limit IκB kinase activity and reduce NF-κB nuclear translocation, potentially explaining why some CAP protocols yield simultaneous antioxidant gene induction and dampened proinflammatory signaling (84–86), ii) Mitogen-activated protein kinase (MAPK)-dependent (particularly p38 and c-Jun N-terminal kinase (JNK)) priming of NLRP3 inflammasome assembly, suggesting that CAP-induced MAPK activation may serve as an upstream licensing signal for subsequent inflammasome-dependent IL-1β maturation (63), iii) Phosphoinositide 3-kinase-Rho family-alpha serine/threonine-protein kinase (PI3K-AKT)-mediated stabilization of HIF-1α under hypoxic or pseudohypoxic conditions, which may be relevant in wound and tumor microenvironments where CAP is applied (86), and iv) mitochondrial ROS acting as a shared upstream input converging on NF-κB, MAPK, and NLRP3 activation simultaneously, potentially explaining coordinated multi-pathway responses to a single CAP exposure (**Figure 3**) (87). We emphasize that this crosstalk network remains an area of active investigation, and most specific interaction points described here have not yet been directly mapped in CAP-treated immune cells. Their inclusion is intended to guide future mechanistic studies rather than to represent established CAP-specific findings.

CAP has been reported to oxidize GSH, perturb mitochondrial potential in primary human immune cells, and activate caspase-3-dependent apoptosis at higher doses (88). In macrophages, CAP-derived ROS have been reported to enhance phagocytic killing (e.g., *Staphylococcus aureus*) and in some experimental contexts, to skew differentiation toward tissue-repairing M2 phenotypes *via* ROS-dependent mechanisms (3, 73). Importantly, the literature describes divergent outcomes of CAP-induced polarization toward either proinflammatory M1-like or anti-inflammatory M2-like states. Several factors appear to influence this polarization outcome: i) Plasma source and RONS composition, ii) Dose and exposure duration (lower cumulative doses and shorter exposure times are more commonly associated with pro-repair M2-like shifts, whereas higher doses more frequently drive proinflammatory M1-like activation or cytotoxicity), iii) Treatment mode (direct CAP exposure *versus* plasma-treated liquid (PTL) treatment can differentially engage surface receptors *versus* bulk cytoplasmic redox sensing), and iv) Tissue/microenvironmental context (local antioxidant capacity, the presence of other immune or stromal cells, and the preexisting inflammatory state of the tissue can modify the net polarization response to a given CAP exposure).

CAP can also induce oxidative post-translational modifications that underlie immunogenic cell death, DC maturation, and M1-like macrophage polarization, with reported downstream effects on tumor-specific T-cell responses (19, 30, 89). Molecular dynamics simulations have suggested that oxidation of programmed death-ligand (PD-L) 1/PD-L2 (and CD47 in related work) may weaken their binding to PD-1/signal regulatory protein α (SIRPα), potentially reducing inhibitory checkpoint signaling (90). Notably, *in vivo* studies have reported that low-dose RONS can paradoxically upregulate PD-1/PD-L1 and favor tumor immune escape, underscoring the strong dose-dependence of CAP’s net immunological effect and the risk of over-generalizing from single-dose or single-model studies (62). However, we emphasize that this evidence derives primarily from computational modeling, and direct experimental validation of these specific oxidative modifications and their functional consequences in CAP-treated biological systems remains limited to a small number of studies. This finding should therefore be regarded as a plausible hypothesis rather than an established mechanism.

Given the apparently contradictory reports of CAP promoting both immune activation and immune suppression, a unifying conceptual framework is useful for interpreting the literature. We propose that the net outcome is governed by the interaction of three variables: i) delivered RONS dose, ii) exposure duration/frequency (single exposure *versus* repeated exposure), and iii) local antioxidant reserve capacity of the target cell/tissue, which determines the effective redox threshold. At doses that transiently increase RONS without overwhelming antioxidant buffering capacity, redox-sensitive signaling tends to favor immune activation, enhanced phagocytosis, immunogenic cell death, and DC maturation. As dose or cumulative exposure increases and antioxidant capacity becomes progressively depleted, cells shift toward either an adaptive, tolerogenic response (e.g., checkpoint upregulation, M2 skewing) as a protective mechanism, or, at still higher doses, toward cytotoxicity and apoptosis/necrosis.

CAP can attenuate immune-mediated inflammation by restoring redox balance, downregulating proinflammatory cytokines, shifting the M1/M2 states of macrophages, and modulating Treg and Th17 responses in models of psoriasis and atopic dermatitis (3, 91, 92). Reviews and hypothesis-driven analyses propose CAP as a redox-modulatory tool that can break cycles of oxidative/reductive stress driving chronic inflammation, autoimmunity, and cancer (78, 92, 93).

Innate immune cells can be exposed to CAP-derived RONS, either directly through topical applications (94) or indirectly through soluble mediators generated in plasma-treated liquids (PTLs) (23, 95, 96). These two treatment modes differ mechanistically in several important respects. Direct CAP exposure delivers a complex mixture including short-lived reactive species, charged particles, transient electric fields, and UV/VUV photons directly to the cell or tissue surface, and may additionally involve physical effects of the electric field on membrane permeability (electroporation-like effects) that are absent in PTL treatment. PTL treatment, by contrast, involves pre-generation of long-lived secondary species in a liquid medium, which is subsequently applied to cells or tissue. This approach delivers a comparatively narrower and more stable RONS profile. Consequently, PTL treatment is generally considered more reproducible and easier to standardize/quantify (e.g., *via* H_2_O_2_ and nitrite/nitrate assays) but may underrepresent certain biological effects attributable to direct exposure. However, reported immune cell responses to PTL, including monocyte/macrophage redox-dependent phenotypic shifts and modulation of neutrophil activation markers, are generally consistent in direction with those observed following direct CAP exposure, though effect magnitude and kinetics can differ, and few studies have directly compared both modalities within the same experimental system (23, 24, 94–97).

Redox signaling constitutes a central control layer for immune cell activation, metabolism, and fate, and CAP represents a tunable external source of RONS capable of engaging these pathways directly (**Figure 3**). Current evidence suggests that, at appropriate doses, CAP-induced redox changes can enhance phagocytic killing, promote immunogenic tumor cell death, and rebalance inflammatory responses. At suboptimal or excessive doses, they may instead reinforce immune checkpoints, promote tolerance, or induce cytotoxicity. A more precise, quantitative understanding and controlled modulation of CAP-driven redox signaling in specific immune cell subsets and tissue microenvironments will be critical for the safe and effective application of this technology in oncology and immune-mediated diseases.

## CAP-induced polarization and phenotypic reprogramming of macrophages

Macrophages dynamically adjust their activation state in response to environmental signals, rather than adopting discrete, mutually exclusive phenotypes (98, 99). The “M1” and “M2” nomenclature, while widely used and retained here for consistency with the cited literature, represents simplified poles of this spectrum and should be understood as “M1-like” and “M2-like” activation states rather than fixed cellular identities.

Classically activated (M1-like) macrophages are characterized by upregulation of surface markers CD80, CD86, and MHC-II. They also exhibit elevated transcription of nitric oxide synthase (NOS) 2, IL-6, IL-12β, and C-X-C motif chemokine ligand (CXCL) 10, robust production of proinflammatory cytokines (TNF-α, IL-1β, IL-6, IL-12, and IL-23), inducible nitric oxide synthase (iNOS) expression, high glycolytic flux, and generation of RONS (100–105). Alternatively activated (M2-like) macrophages, by contrast, rely predominantly on oxidative phosphorylation and express CD206, CD163, and arginase-1 (Arg-1), supporting anti-inflammatory and tissue-regenerative functions (106–109).

**Figure 4:**
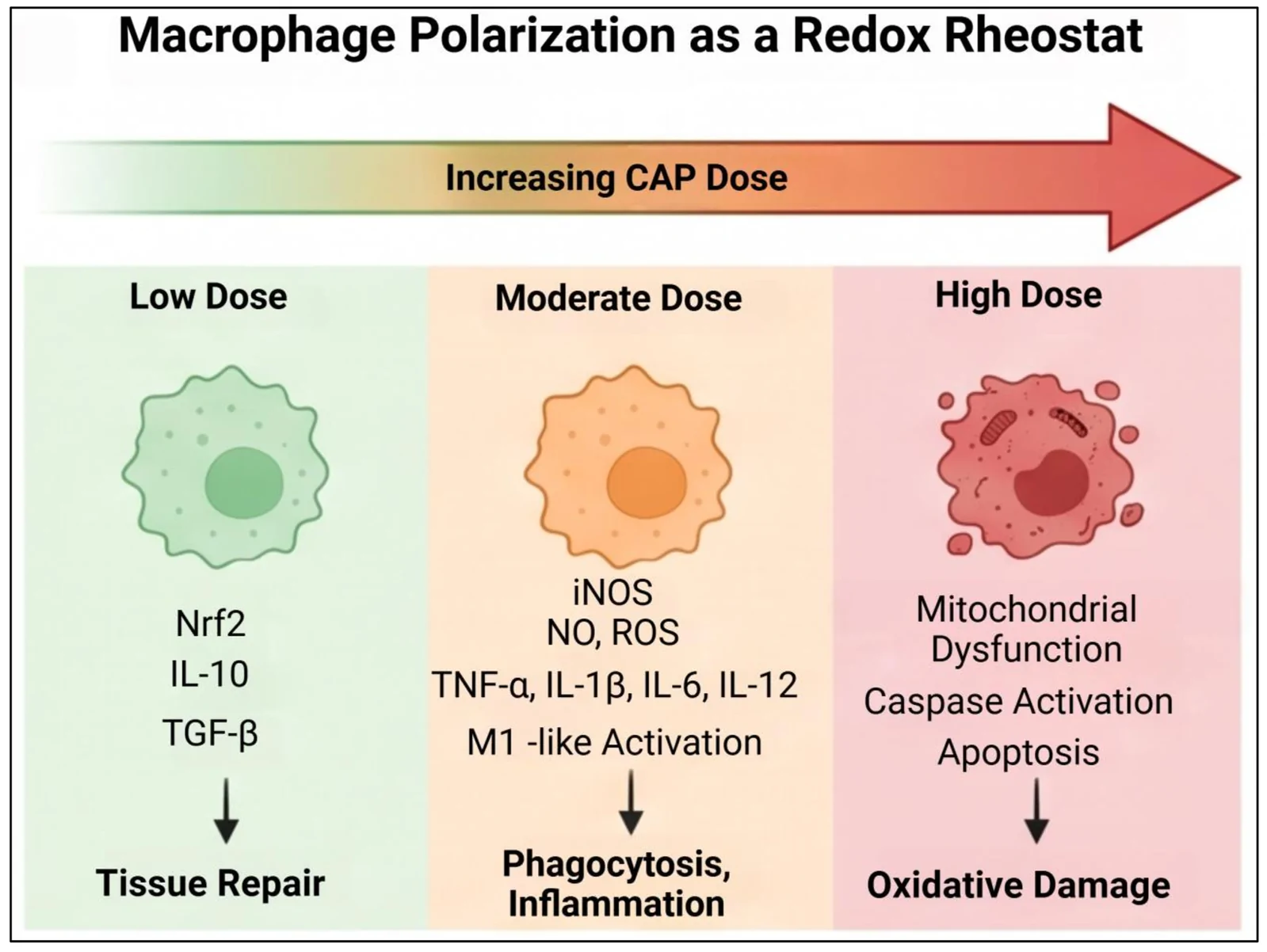
CAP as a redox rheostat of macrophage polarization and function. CAP modulates macrophage phenotype in a dose-dependent manner, promoting reparative, inflammatory, or cytotoxic outcomes depending on redox intensity. Low CAP exposure may induce adaptive redox signaling, Nrf2 activation, and tissue remodeling. Moderate CAP exposure can promote M1-like immune activation characterized by increased iNOS, nitric oxide, ROS, TNF-α, IL-1β, IL-6, IL-12, phagocytosis, and antimicrobial activity. Excessive CAP exposure can overwhelm antioxidant defenses, leading to mitochondrial dysfunction, lipid peroxidation, caspase activation, apoptosis or necrosis, and reduced macrophage viability.

Multiple studies have shown that CAP exposure can shift human monocyte-derived macrophages and murine bone marrow-derived macrophages (BMDMs) from an M2-like toward an M1-like state (**Figure 4**, **Table 2**) (104, 110, 111). In an *in vitro* study, argon-based kINPen jet treatment of murine BMDMs induced significant upregulation of iNOS, TNF-α, and IL-6, alongside suppression of CXCL1, monocyte chemoattractant protein (MCP)-1, and M2-associated markers in a dose-dependent manner (110). Likewise, CAP-treated THP-1-derived macrophages exhibited increased CD86 (M1) relative to CD163 (M2), enhanced TNF-α and IL-12 secretion, and an anticancer secretome, collectively suggesting predominant M1-like activation (104). *Ex vivo* primary rat macrophages exposed to CAP upregulated IL-6 and IL-12 and showed improved antigen-presenting capacity, which was associated with amplified T cell effector function and enhanced antitumor responses when translated to an *in vivo* tumor model (112).

**Table 2:** Redox-driven immunomodulation of macrophages.

Plasma source	Treatment parameters	Macrophage model	Principal findings	Proposed mechanisms	Reference
Micro-DBD plasma device	Exact exposure settings not provided	THP-1 monocytes differentiated toward macrophages	Increased CD86 expression and favored stronger M1-skewed differentiation with antitumor effects on glioma	Immune modulation through ROS/RNS-linked differentiation, cytokine induction, and TNF-α/IL-12-mediated tumor cell killing	(104)
Plasma-treated medium (source not specified)	Indirect exposure of macrophages to oxidized medium	Murine bone marrow-derived macrophages, including TAM	Increased NOS2/iNOS in M0 and TAM conditions and shifted cytokines toward TNF-α, IL-6, IL-12, CCL4, and CXCL9, while reducing MCP-1 and CXCL1	Redox-sensitive macrophage plasticity without a fully robust binary M1/M2 switch	(110)
kINPen non-thermal atmospheric pressure plasma jet	Short exposures activated MEK-ERK; longer exposures activated JNK and caspase-3 (exact durations not provided)	Primary human monocytes	Time-dependent shift with early activation of proliferative pathways followed by increased apoptotic signaling	Differential MAPK control, with MEK/ERK supporting stimulation after short exposure and JNK/caspase-3 supporting apoptosis after longer exposure	(111)
Atmospheric pressure argon plasma jet; plasma-treated medium therapy	Repeated intraperitoneal application of plasma-treated medium in tumor-bearing mice	Murine pancreatic tumor-infiltrating macrophages *in vivo*	Treatment reduced tumor burden, increased macrophage infiltration, and decreased CD206+ M2-like macrophages; T cells and calreticulin staining also increased	Immunomodulation likely involves reduced M2-associated macrophage states and immunogenic cancer cell death signals	(113)

Beyond direct CAP exposure, M1 polarization has also been demonstrated in combination therapy settings using *in vivo* murine tumor models. CAP-activated macrophages displayed an inflammatory transcriptional signature consistent with M1 polarization and enhanced antigen presentation. These macrophages also promoted downstream CD8^+^ T cell activation in murine tumor models (113). In a separate murine tumor model, treatment with trehalose + CAP repolarized tumor-associated macrophages from an immunosuppressive M2-like toward a proinflammatory M1-like phenotype. This reversed immunosuppression within the tumor microenvironment and enhanced tumor antigen presentation to T cells (19).

Notably, M1 skewing is not a universal outcome of CAP treatment, and several studies report divergent, M2-favoring responses. In wound-healing models, lower doses of CAP or plasma-activated liquid (PAL) promote M2-like phenotypes characterized by enhanced oxidative phosphorylation, fatty acid oxidation, anti-inflammatory signaling, and growth factor secretion, which collectively support resolution of inflammation and enhance tissue repair (114–118). Consistent with this, *in vitro* human monocyte-derived macrophages exposed to CAP at defined parameters and short treatment durations demonstrated a temporal decrease in M1 markers and a reciprocal increase in M2 markers, confirming that certain parameter combinations selectively favor anti-inflammatory reprogramming (27).

However, these findings appear contradictory, as CAP is reported to drive both pro-inflammatory M1-like and anti-inflammatory M2-like polarization, sometimes using superficially similar treatment approaches (**Table 2**). Several factors likely contribute to this apparent inconsistency and should be considered carefully when interpreting the literature. First, macrophage source and species differ substantially across studies (murine BMDMs, human THP-1 cell line, primary rat macrophages, tumor-associated macrophages *in situ*), and baseline activation state and species-specific redox sensitivity may strongly influence polarization outcome. Second, dose and treatment duration vary considerably and are inconsistently reported using comparable metrics, making it difficult to determine whether apparently divergent studies truly represent different points along the same dose-response curve or reflect device-specific differences. Third, readout timing differs across studies (acute *versus* delayed assessment), and given that macrophage polarization can be transient and evolve over time following redox stress, single time-point measurements may capture different phases of a dynamic response. Fourth, tissue microenvironments differ markedly between tumor models (where baseline immunosuppression and hypoxia predominate) and wound-healing models (where baseline tissue repair signaling predominates), potentially predisposing the same CAP dose to different net outcomes depending on the microenvironmental context. We recommend that future studies explicitly report macrophage source, precise dosimetry, and multiple readout time points to enable more direct cross-study comparison and resolution of these apparent discrepancies.

Accumulating evidence suggests that CAP-mediated macrophage polarization depends on exposure duration, plasma source (DBD *versus* APPJ), gas composition, and dose (**Figure 4**), consistent with the device-chemistry relationships discussed earlier in this review. Although not universal, high oxidative stress frequently favors M1 skewing and immune activation, plausibly *via* robust NF-κB activation and HIF-1α-driven glycolytic reprogramming. Conversely, lower oxidative stress sustains M2-like polarization, potentially through predominant Nrf2-driven antioxidant gene induction without sufficient NF-κB engagement. Prolonged or excessive exposure, however, may lead to oxidative overload, mitochondrial dysfunction, and loss of function or viability rather than productive polarization in either direction (30, 99, 119). This dose-response relationship positions CAP as a potentially tunable immunomodulatory tool rather than a nonspecific inflammatory stimulus, though we emphasize that the distinct physicochemical outputs governing this transition remain incompletely defined and likely vary by macrophage source and tissue context. Currently, no universally adopted dosimetry standard exists for CAP-related immune cell studies. Several proposed approaches offer partial solutions, including reporting of energy density delivered per unit area (J/cm²) and chemical dosimetry based on measured H_2_O_2_-equivalent or nitrite/nitrate concentration in treated liquids. Addressing these limitations will require coordinated efforts to standardize device reporting, validate dose-response relationships in human-relevant models (e.g., human tissue explants, organoids), and develop clinically applicable methods for assessing macrophage polarization states *in vivo*.

It is important to note that the great majority of the evidence discussed above derives from *in vitro* and *ex vivo* cell culture systems, with a smaller subset of findings extended to *in vivo* murine tumor or wound-healing models. To date, direct clinical evidence for CAP-induced macrophage polarization in human patients is essentially absent, and the translational relevance of the *in vitro* and murine findings summarized here therefore remains to be established. Several factors are likely to complicate future clinical translation. The substantial variability in plasma device architecture and operating parameters described above means that treatment protocols validated with one device/dose combination may not directly translate to another, even when nominally targeting a similar macrophage-polarization endpoint. Human tissue and immune contexts present greater cellular and microenvironmental heterogeneity than controlled *in vitro* systems or inbred murine models, potentially altering dose-response relationships.

## Dendritic-cell maturation, cross-presentation, and trafficking

**Figure 5:**
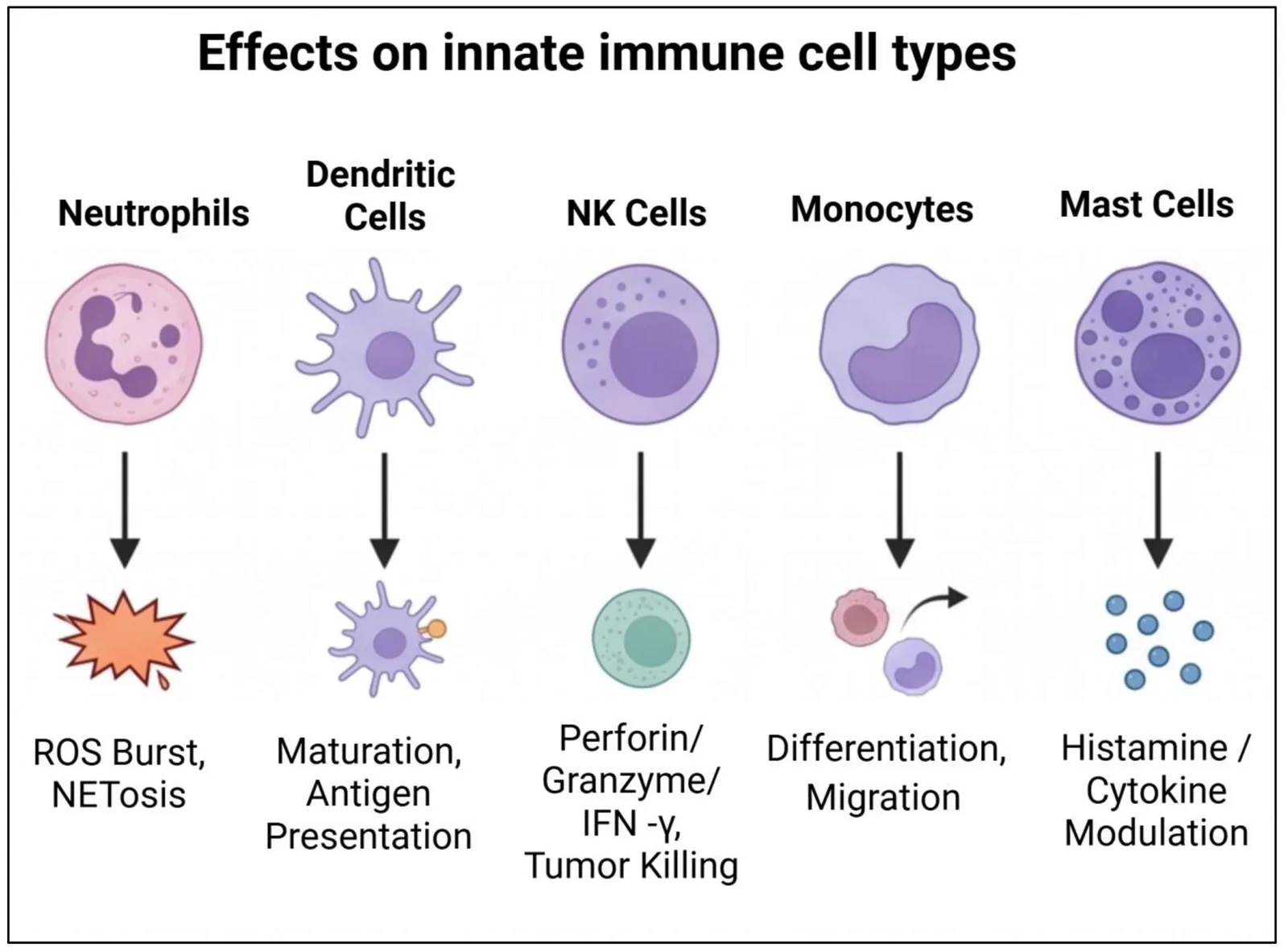
Innate immune cell-specific responses to CAP-derived RONS. CAP-derived RONS influence neutrophils, dendritic cells, NK cells, monocytes, and mast cells, thereby shaping innate immune activation, antimicrobial defense, and immune regulation. CAP may influence neutrophils, resulting in respiratory burst, ROS production, degranulation, and NETosis. In dendritic cells, CAP-associated redox signaling can modulate maturation, antigen presentation, MHC-I/MHC-II expression, co-stimulatory molecules such as CD80, CD86, and CD40, and cytokine production, thereby linking innate and adaptive immunity. In natural killer cells, CAP may enhance cytotoxicity, perforin and granzyme B release, IFN-γ production, and tumor cell recognition, especially in the context of immunogenic cancer cell death and DAMP release. Mast cells and other innate immune cells may contribute to CAP-mediated regulation of histamine release, inflammatory mediators, skin immunity, and wound responses.

DCs are the primary antigen-presenting cells that connect innate and adaptive immunity. Their maturation program (i.e., upregulation of co-stimulatory molecules, cytokine production, and cross-presentation capacity) critically shapes whether T cell responses become tolerogenic or immunogenic (120, 121). Current evidence suggests that CAP can act as a non–pathogen-associated molecular pattern (PAMP)-dependent stimulus for DC maturation and migratory programming (**Figure 5**), with reported effects on activation marker expression, cytokine release, and antigen presentation directly or indirectly in cancer and inflammatory disease contexts, including modulation of CD80, CD83, CD86, MHC class II, CD40, and IL-12/IL-10 production (17, 31, 122). In human monocyte-derived DCs (moDCs), argon-based CAP increased activation markers CD25, CD40, CD83, and proinflammatory cytokines IL-1α, IL-6, IL-23, facilitating T cell co-stimulation and potential antitumor immunity (17). In contrast, N_2_-plasma-treated liquid (indirect CAP) suppressed TNF-α/LPS-induced activation markers CD80, CD86, MHC-II and reduced IL-6 in mouse bone marrow-derived dendritic cells (BMDCs), indicating a DC-dampening and anti-inflammatory effect in a murine model of psoriasis (123). DCs also play a central role in the cancer immunity cycle, serving as mediators between tumor antigen capture and adaptive immune activation. In a melanoma mouse model, CAP treatment increased CD103^+^ DC infiltration, thereby enhancing antigen presentation and initiating adaptive immune responses. Following CAP treatment, DCs migrated to draining secondary lymphoid organs, including the tumor-draining lymph nodes (TDLNs) and the spleen. This migration is essential for presenting tumor antigens to T cells and activating them for anticancer immunity (124). In a separate *in vitro* study, DCs enhanced antigen cross-presentation when exposed to CAP-treated tumor lysates, resulting in more robust CD8 T-cell priming (122).

In a combinatorial approach (*in vivo* murine tumor model), the natural disaccharide (trehalose) potentiated CAP-induced immunogenic cell death by enhancing eIF2α phosphorylation, a critical upstream event in the ER stress-mediated damage-associated molecular patterns (DAMP) release cascade. Delivery of CAP + trehalose *via* an injectable hydrogel system promoted DC maturation and initiated CD8 T cell-mediated antitumor immune responses (19).

In contrast to the activating effects described above, N_2_-plasma-treated liquid (indirect CAP) suppressed TNF-α/LPS-induced activation markers CD80, CD86, MHC-II and reduced IL-6 in mouse BMDCs, indicating a DC-dampening and anti-inflammatory effect in a murine model of psoriasis (123). Across the models reviewed, CAP modulates DC function either directly or *via* induction of immunogenic tumor cell death, with reported effects ranging from DC maturation, co-stimulatory ligand upregulation, and proinflammatory cytokine release supporting antitumor immunity, to dampened DC activation and reduced effector T-cell differentiation in models of inflammatory skin disease. The overall DC outcome is highly context- and formulation-dependent. We propose that this divergence is best explained by several variables rather than representing a true inconsistency in CAP’s mechanism of action. For instance, treatment modality differs fundamentally between the activating and suppressive studies. Direct argon-CAP exposure activated DCs, whereas the suppressive effect was observed using nitrogen-plasma-treated liquid, an indirect exposure route delivering predominantly long-lived RNS species (as discussed in the Reactive Species section). Another factor that may contribute is the disease/microenvironmental context and, lastly, the DC baseline activation state (TNF-α/lipopolysaccharide (LPS)-prestimulated DCs *versus* unstimulated or tumor-antigen-loaded DCs). This activation pattern is conceptually consistent with the dose- and context-dependent threshold model proposed in the Molecular Pathways section (**Figure 3**), suggesting that DC responses to CAP may follow similar underlying principles to those described for macrophage polarization, though this has not been directly tested in side-by-side comparative studies.

## Neutrophil immunomodulation

Cold atmospheric plasma strongly affects innate immunity, and neutrophils are among the best-studied targets (**Figure 5**). The current evidence base is derived predominantly from preclinical studies (*in vitro* human cells and animal models) and demonstrates that CAP induces neutrophil activation and functional reshaping without necessarily causing cell death (125), in line with the broader theme of this review that CAP engages redox-sensitive signaling rather than acting solely as an indiscriminately cytotoxic stimulus. Direct CAP exposure or plasma-treated solution has been shown to reduce the time to peak ROS production in human neutrophils, alter migration in a time-dependent manner, and enhance the expression of surface integrins and selectins (e.g., CD11b, CD62L), consistent with an activated phenotype, without inducing apoptosis (21).

In a 3D upper respiratory tract (URT) model, polymorphonuclear neutrophils (PMNs) isolated from human blood were exposed to CAP, resulting in modulation of intracellular ROS, migration, formation of neutrophil extracellular traps (NETs), and surface CD11b/CD62L/CD66b expression. Notably, this study directly compared two device types operating at different RONS output levels, a high-RONS plasma-intensive care device (PIC) and a milder nebulized plasma (NP) device, without evidence of overt cellular damage or overstimulation with either device (23). In summary, a tailored CAP treatment approach could be used to reduce microbial loads in the upper respiratory tract while maintaining, rather than overwhelming, the immune response, offering a non-antibiotic approach to managing ventilator-associated pneumonia (VAP). However, this specific therapeutic application remains a proposal based on an *in vitro* tissue model and has not yet been tested in an *in vivo* infection model or clinical setting.

In a murine dermal wound model, CAP accelerated wound healing and was associated with early recruitment of neutrophils to the wound site alongside redox-regulated signaling involving Nrf2 and p53 (126). This finding extends the *in vitro* activation/migration data to a physiologically relevant *in vivo* tissue-repair context, though the precise contribution of neutrophil recruitment (*versus* other CAP-induced effects) to the observed healing was not isolated in this study.

Beyond activation and migration, CAP treatment has been reported to primarily affect neutrophil function by inducing robust NET formation and increasing IL-8 release, rather than by altering direct bactericidal capacity. CAP-induced NET formation was reported to be comparable in kinetics and magnitude to that induced by phorbol 12-myristate 13-acetate (PMA). Unlike PMA, CAP treatment did not induce early apoptosis and preserved bactericidal activity against *Pseudomonas aeruginosa* and *S. aureus*. This NETosis was reported to depend specifically on short-lived plasma species (consistent with the primary/secondary species distinction discussed in the Reactive Species section) and was accompanied by increased IL-8 release, suggesting amplified neutrophil recruitment and local inflammatory signaling at treated sites (125).

Collectively, CAP acts as a tunable modulator of neutrophil function, capable of enhancing ROS production, migration, and NET formation while generally preserving bactericidal capacity and avoiding overt cytotoxicity at the doses tested. However, the same NET-inductive property that supports antimicrobial defense also carries a context-dependent risk of amplifying local inflammatory signaling (*via* IL-8 release and NET-associated tissue effects), particularly under repeated or high-dose exposure conditions. Defining the precise dose and treatment frequency boundaries that separate beneficial neutrophil activation from potentially harmful excessive NETosis represents an important direction for future research.

## Natural killer cell activation

NK cells are innate lymphocytes that are central to cancer immunosurveillance (127). Because they can recognize and eliminate tumor cells without prior antigen sensitization, NK cells are attractive targets for immunotherapeutic strategies (128, 129). Compared with the other innate immune cell types discussed in this review, however, the evidence addressing CAP’s effects on NK cells is considerably more limited, and largely restricted to a small number of studies.

In one *in vitro* study, treatment of skin cancer cells (rather than exposing NK cells directly) with non-thermal plasma (NTP, used synonymously with CAP) increased tumor cell susceptibility to NK cell-mediated killing (**Figure 5**). Plasma-treated skin cancer cells exhibited time-dependent cytotoxicity and altered expression of activating and inhibitory NK cell ligands. This resulted in significantly enhanced NK cell-mediated killing of plasma-treated tumor cells, as well as increased IL-6 and IL-8 release, consistent with a proinflammatory microenvironment (130). These findings suggest that cold plasma can act as an immunogenic sensitizer of tumor cells, indirectly strengthening NK antitumor activity by making cancer cells more susceptible targets, supporting CAP as a potential adjuvant to NK cell-based immunotherapies. Consistent with these *in vitro* findings, increased NK-associated antitumor activity was also observed *in vivo* following local NTP treatment of melanoma in a murine model, suggesting that CAP may influence not only tumor cells but also the broader immune context governing NK effector function (124). Together, these studies support a preliminary model in which CAP may promote antitumor immunity, at least in part, by enhancing tumor cell susceptibility to NK cell-mediated cytotoxicity and by reshaping local conditions to favor innate immune effector functions. However, several important limitations should be explicitly noted. The available data are drawn from a very small number of studies within a single tumor type. Furthermore, most available data focus on indirect effects, such as how CAP conditions tumor targets or the surrounding microenvironment. Nevertheless, the direct effects of cold plasma on NK cell viability, phenotype, trafficking, and cytotoxic function remain incompletely characterized and will therefore be an important next step for understanding and optimizing CAP-based immunotherapies.

## Antiviral effects and immune potentiation

CAP exerts antiviral activity through two partially overlapping mechanisms: direct virion inactivation and potentiation of host immune responses. RONS generated by CAP or plasma-activated medium damage viral envelopes, surface proteins, and RNA, reducing the infectivity of SARS-CoV-2–like pseudovirus (131), SARS-CoV-2, influenza H1N1 (132), HSV-1 (133, 134), and coronaviruses such as HCoV-229E (135). In HCoV-229E-infected MRC-5 cells, CAP-treated medium reduced viral replication and attenuated inflammatory signaling through suppression of NF-κB and MAPK pathways (135).

Beyond direct inactivation, it has been proposed that CAP-triggered DAMP release promotes antigen-presenting cell (APC) recruitment, phagocytosis, and the production of cytokines including IFN-γ, IL-6, TNF-α, and IL-1β, thereby enhancing immune responses against viral targets (89, 136, 137). It is important to clarify that mechanistic evidence for DAMP-mediated immune activation comes predominantly from CAP/NTP cancer ICD studies, while viral evidence is preclinical and limited mostly to latent HIV cell models, with *in vivo* infection-model confirmation still pending.

These effects are not universal. Certain adenovirus and bovine herpesvirus strains have shown neutral or even enhanced infectivity following CAP exposure, underscoring the need for careful dosing optimization and mechanistic studies (132, 138). Much of the reported variability in antiviral outcomes mentioned above likely reflects underlying differences in plasma treatment parameters (as discussed in earlier sections). Gas composition strongly influences the relative ROS/RNS output, with ROS-dominant chemistries (e.g., He/Ar-fed jets) expected to favor direct oxidative damage to viral envelope lipids and capsid proteins. Exposure time and applied power directly determine cumulative RONS dose delivered to the virus or virus-infected cell, and insufficient dose may fail to achieve the oxidative threshold required for envelope disruption or genome damage, potentially explaining reports of neutral efficacy against certain virus strains. Additionally, structural differences among viruses themselves, for example, enveloped *versus* non-enveloped viruses, likely modulate intrinsic susceptibility to RONS-mediated damage, independent of treatment parameters. Systematic studies directly comparing standardized RONS doses across multiple virus structural classes are currently lacking.

The antiviral potential of CAP is real but context-dependent. Direct virion inactivation through RONS-mediated oxidative damage is reasonably well supported, whereas immune potentiation *via* DAMP release and cytokine activation is less predictable and has not been demonstrated consistently across virus types or model systems. Several specific knowledge gaps must be addressed before CAP can be considered a reliable antiviral therapeutic strategy: i) direct confirmation of DAMP-mediated immune potentiation in viral infection models, ii) dose-standardized comparison across virus structural classes (enveloped vs. non-enveloped, RNA vs. DNA viruses) to distinguish device/parameter-dependent effects from virus-intrinsic susceptibility, iii) mechanistic clarification of why certain viruses (e.g., adenovirus, bovine herpesvirus) show neutral or enhanced infectivity following CAP exposure, iv) *in vivo* validation of antiviral efficacy and immune potentiation, as the evidence summarized here derives predominantly from *in vitro* cell-culture and cell-free virus inactivation studies, with essentially no clinical data currently available, and v) standardized dosimetry reporting specific to antiviral applications, given that treatment parameters likely account for much of the inter-study variability discussed above. Addressing these gaps will be essential for determining whether CAP can be reliably positioned as an adjunct antiviral or immune-potentiating therapeutic strategy.

## Knowledge gaps, technological needs, and future directions

**Figure 6:**
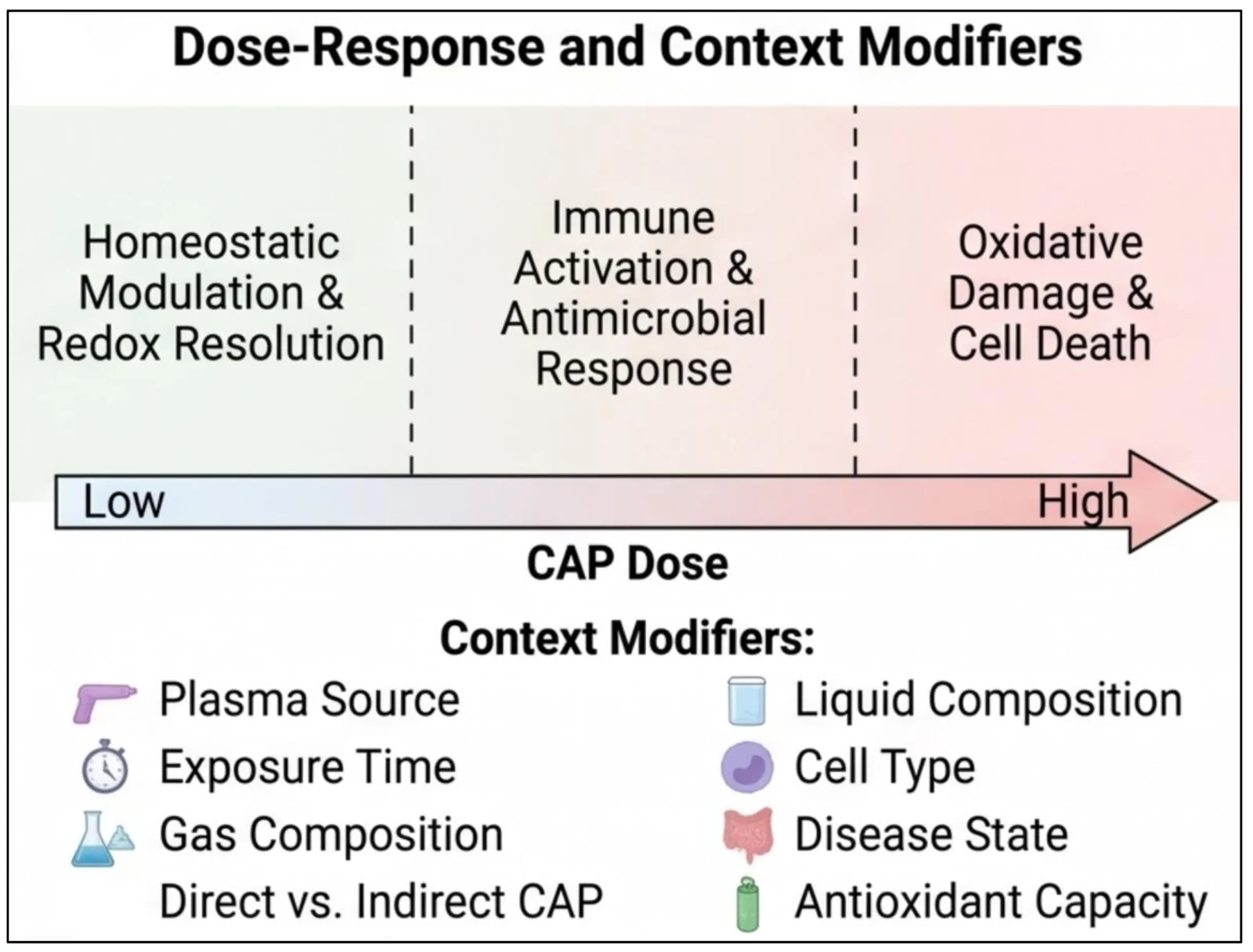
Dose- and context-dependent immunomodulation by CAP-derived RONS. CAP produces dose-dependent biological effects that range from homeostatic redox modulation to immune activation and, at excessive exposure, oxidative damage and tissue injury. Low CAP doses may support redox resolution and tissue homeostasis, whereas moderate doses can activate immune and antimicrobial responses. High CAP doses may overwhelm antioxidant defenses, leading to oxidative injury, mitochondrial dysfunction, and cell death. CAP outcomes are influenced by plasma source, exposure time, gas composition, direct versus indirect CAP treatment, liquid composition, cell type, disease state, and local antioxidant capacity. Together, these variables determine whether CAP-induced redox signaling remains adaptive and therapeutic or becomes cytotoxic.

While CAP holds promise as an immunomodulatory platform across oncology, wound repair, infection control, and inflammatory disease, substantial mechanistic gaps remain. As detailed above, device heterogeneity (plasma jets *versus* dielectric barrier discharge), inconsistent dosing parameters, limited tissue penetration (typically < 1 mm for most devices), and the poor integration of CAP with existing delivery systems collectively restrict clinical applicability (**Figure 6**) (139).

Several strategies may help address these limitations. Plasma-activated hydrogels could extend reactive species delivery beyond superficial tissues (140), while computational modeling, machine-learning-assisted dose optimization, and multi-omics profiling may bring greater mechanistic clarity to the dose-outcome relationships (50). Safety, however, remains insufficiently defined with respect to genotoxicity, off-target effects, and the consequences of repeated CAP exposure (30, 139–141).

Despite recent advances, several specific limitations constrain the strength of conclusions that can currently be drawn from the CAP-immunology literature. The predominance of *in vitro* (**Table 3**) and murine *in vivo* (**Table 4**) studies and the absence of clinical validation limit current evidence. Many foundational studies cited in this review employ small sample sizes, limited study designs, or limited biological replicates, raising concerns about statistical power, effect-size reliability, and generalization across human population variability (e.g., donor-to-donor differences in baseline antioxidant capacity, as discussed in the Molecular Pathways section).

**Table 3:** Selected *in vitro* studies on CAP-induced immune modulation.

Immune cell model(s)	CAP modality/approach	Main immunomodulatory findings	References
Leukocytes	Direct cold plasma	Altered microparticle release/composition; changes in proteins related to immune signaling and redox homeostasis	(143)
Human monocytes, monocyte-derived macrophages (MDMs)	Direct CAP exposure	Increased ROS and reduced viability in monocytes; MDMs polarized toward anti-inflammatory M2 phenotype	(27)
U937 monocytes/macrophage-like cells	Direct CAP + PMA stimulation	Enhanced differentiation *via* NF-κB activation; increased TNF-α and IL-6 secretion	(144)
Human monocytes → macrophages; co-culture with tumor cells	Micro-DBD device, direct CAP on monocytes	Drives differentiation toward M1-like, antitumor macrophages; CAP-stimulated macrophages secrete anticancer cytokines and suppress metastatic solid cancer cell proliferation and stemness	(104)
NK cells co-cultured with skin cancer cells	kINPen plasma jet	Enhanced NK cell killing activity against tumor cells; upregulation of activating/inhibitory receptors	(130)
Human granulocytes (PMNs)	Direct and indirect CAP treatment	Enhanced integrin/selectin expression, altered migration, increased ROS/RNS production—indicative of increased immunoreactivity	(21)
Human monocyte-derived dendritic cells (moDCs)	Argon plasma jet	Upregulation of CD25, CD40, CD83; increased IL-1α, IL-6, IL-23 secretion	(17)
THP-1 monocytes	Direct CAP irradiation	Suppressed IL-6 and inflammatory gene expression; Nrf2 nuclear translocation implicated in anti-inflammatory response	(14)
THP-1 and RAW 264.7 macrophages	Plasma-activated liquid	PAL promotes M1 polarization *via* ROS/JAK2/STAT1; PAL-treated macrophages inhibit melanoma cell proliferation	(145)
RAW 264.7 macrophages; gingival fibroblasts	Homemade CAP jet, repeated applications	Time- and dose-dependent proinflammatory activation of macrophages (altered morphology and cytokine release at longer/repeated exposures; fibroblasts show pro-healing responses)	(146)
Human PMNs in 3D upper airway model	Two air-based CAP devices, 5 min	Modulates migration, ROS, NETosis, and activation markers without overt damage or overreaction; one device shows milder functional impact but also lower RONS/antibacterial effect	(23)

**Table 4:** Selected *in vivo* studies on CAP-induced immune modulation.

Model/system	CAP intervention	Main immune modulation outcomes	References
Mouse (B16-F10 melanoma bilateral model: one leg treated, the other observed for abscopal effect)	Nanosecond pulsed streamer discharge applied to primary tumor site	Suppressed growth at the untreated distant site (“abscopal” effect); increased inflammatory cytokines from splenocytes suggest both innate/adaptive immunity activation	(147)
Mouse vaccination/tumor challenge model with OVA antigen	Gas plasma-modified protein vaccination	Enhanced T cell activation, IFN-γ production, increased APCs, reduced tumor burden, and immunomodulatory potential *via* ROS/RNS modifications	(148)
Mouse (CT26 tumor)	CAP-activated hydrogel with nanoadjuvant	Enhanced dendritic cell activation; increased CD4+ and CD8^+^ T cells; complete tumor ablation and survival upon rechallenge	(149)
Mouse (spinal cord injury)	CAP-activated saline for 14 days postinjury	Reduced proinflammatory macrophage infiltration; decreased IL-1β, IL-6, TNF-α; improved motor function recovery	(150)

Reproducibility is further constrained by variation in plasma devices, exposure parameters, and RONS quantification (as detailed extensively in the CAP Generation and Reactive Species sections), while optimal dosing windows for immune activation *versus* suppression remain undefined. As discussed earlier, direct CAP exposure and PTL treatment differ mechanistically in the physicochemical stimuli delivered. Yet, it remains unclear to what extent mechanistic findings obtained with one modality generalize to the other, complicating interpretation of the broader literature and the selection of a treatment approach for specific clinical applications.

Several additional aspects of CAP biology also remain insufficiently explored, including its effects on suppressive compartments such as myeloid-derived suppressor cells (MDSCs), regulatory T cells (Tregs), and stromal barriers and the impact of repeated exposure on mitochondrial integrity, genomic stability, and long-term viability of innate immune cells. There is also insufficient knowledge on the contribution of reactive nitrogen species, particularly peroxynitrite and nitric oxide, to innate immune polarization independently of ROS.

Mechanistically, the central unresolved question is how specific ROS and RNS compositions and exposure conditions determine whether CAP promotes immune activation or suppression, induces immunogenic cell death or tolerance, and supports durable immune memory. No reports could be identified on the influence of device architecture on specific immunological functions. Addressing this will require standardized RONS quantification across device platforms, systematic mapping of CAP-induced redox signaling, epigenetic reprogramming, and post-translational modifications across immune cell subsets, and the use of organ-on-chip and 3D tumor microenvironment models to bridge the *in vitro–in vivo* gap. Additional technological priorities include plasma-activated hydrogels to extend reactive species delivery beyond superficial tissue barriers and endoscopic and implantable CAP platforms for deep-seated tumors. Equally important is rigorous assessment of genotoxicity, mitochondrial damage, off-target oxidative injury, immunotoxicity, and autoimmunity risk following both single and repeated CAP exposure, areas of safety characterization that remain substantially underdeveloped.

Beyond the mechanistic and technological gaps outlined above, several translational barriers must be addressed before CAP-based immunomodulatory approaches can reach clinical practice. Reproducibility across devices and protocols remains limited by the device heterogeneity and dosimetric inconsistency discussed throughout this review. Regulatory pathways specific to CAP-based immunomodulatory (as opposed to antimicrobial or wound-healing) applications remain relatively immature, and clarification of applicable device-approval frameworks will be an important prerequisite for clinical development. Long-term safety data, particularly regarding cumulative effects of repeated CAP exposure relevant to chronic disease applications (e.g., chronic wound care), remain limited. Finally, multicenter validation using standardized dosimetry protocols across independent laboratories, followed by controlled human clinical trials, will be necessary to establish whether the immunomodulatory effects are reproducible in human tissue and clinically meaningful.

Progress will depend on interdisciplinary collaboration among physicists, immunologists, clinicians, and regulatory scientists, with coordinated efforts focused on standardized CAP protocols for multicenter studies and evidence-based regulatory frameworks for device certification, manufacturing standardization, and good manufacturing practice (GMP)-compliant CAP-based combination products.

## Conclusions

CAP lies at the intersection of physics, chemistry, and immunology. It can modulate innate and adaptive immunity in a context-dependent manner, and has been shown to affect macrophage and dendritic cell function, T cell activation, immunogenic cell death, and tissue repair. Across multiple independent *in vitro* and murine studies, CAP has been shown to modulate redox-sensitive signaling pathways, particularly NF-κB and Nrf2-Keap1, in a dose-dependent manner, directly inactivate several enveloped viruses *via* RONS-mediated oxidative damage to viral envelopes and genomes, and induce phenotypic shifts in macrophage polarization and dendritic cell maturation markers under defined *in vitro* and murine conditions.

However, outcomes remain heterogeneous across device types, treatment parameters, and experimental models. The mechanistic basis of key immunological questions, particularly the conditions governing immune activation *versus* suppression, and the durability of any resulting immunological memory, remains largely unresolved.

It is also worth situating CAP within the broader landscape of redox-based therapeutic strategies (e.g., photodynamic therapy, ozone therapy, pharmacological Nrf2 activators). CAP’s distinguishing feature is its capacity to deliver a complex, tunable, multi-species RONS profile alongside physical stimuli (electric fields, charged particles, photons) not present in single-agent redox therapies, a feature that may underlie its broad pathway engagement but also contributes to its dosimetric complexity relative to more chemically defined approaches.

Addressing the multitude of technological, methodological, and regulatory challenges outlined in this review, including standardized dosimetry, expanded human and multicenter validation, and rigorous long-term safety assessment, will be essential for CAP to progress toward a clinically meaningful immunotherapeutic tool. Rather than anticipating an imminent transition, we view the coming decade as an important period for establishing this mechanistic and methodological foundation. As plasma science increasingly converges with systems immunology, biomaterials engineering, and precision medicine, this groundwork may in time support the responsible translation of CAP from an experimental modality toward validated integrative therapeutic strategies.
